# Rhabdomyoma of paracarotid space: case report

**DOI:** 10.3389/fsurg.2026.1839742

**Published:** 2026-05-28

**Authors:** Moysis Moysidis, Anna Zissi, Angeliki Chorti, Katerina Zarampouka, Angeliki Cheva, Theodosios Papavramidis

**Affiliations:** 1 3rd Surgical Department, AHEPA University General Hospital of Thessaloniki, Thessaloniki, Greece; 2 Faculty of Medicine, Aristotle University of Thessaloniki, Thessaloniki, Greece; 3 1st Propaedeutic Department of Surgery, AHEPA University Hospital, Aristotle University of Thessaloniki, Thessaloniki, Greece; 4 Pathology, Istodierevnitiki SA, Thessaloniki, Greece; 5 Department of Pathology, Faculty of Medicine, Aristotle University of Thessaloniki, Thessaloniki, Greece

**Keywords:** case report, head and neck, paracarotid space, rhabdomyoma, thyroid

## Abstract

Rhabdomyoma is a skeletal muscle lesion. It is considered a rare and non-aggressive tumor originating from striated muscle. It may occur in the heart or in extracardiac region. In adults, it most typically occurs in the larynx and pharynx. The occurrence in the paracarotid space is extremely rare, and no similar cases was found in the recent literature. We present an unprecedented case of a paracarotid rhabdomyoma in a 50-year-old female who underwent total thyroidectomy for a goiter. Clinical examination revealed an asymptomatic cervical mass on the left side of the neck. Ultrasound showed a hypoechoic, solid lesion, which was characterized as a parathyroid adenoma. The diagnosis was confirmed postoperatively by histopathology. The aim of this article is to report a rare extracardiac location of this tumor. Complete surgical excision is paramount due to the high potential for recurrence in cases of incomplete excision.

## Introduction

Rhabdomyoma is an extremely rare benign mesenchymal tumor of skeletal muscle that may occur in cardiac and extracardiac locations. The cardiac type is characterized as a hamartomatous lesion and is considered to be one of the most common tumors in infancy, accounting for 50%–70% of pediatric cardiac tumors (1). It often affects boys between birth and the age of three and is frequently associated with other malformations. Extracardiac rhabdomyomas are true neoplasms and can be further divided into three types: fetal, adult and genital.

Fetal rhabdomyomas are usually located in the head and neck region and occur in younger patients (between 25 and 40 years old). The genital type is found in the vulva or vagina in middle-aged women and is classified as such due to the morphological similarities between the types. According to the literature, 77% of all extracardiac-rhabdomyomas are located in the head and neck, and 14% located in the genital region (2). Finally, the adult type occurs in middle-aged adults either as a slowly growing mass, or a multifocal lesion. Both fetal and adult types occur more frequently in males. The male-to-female ratio is 4:1 and the mean age of occurrence is 50 years (3). The present case is rare for various reasons, including gender, age of presentation, differential diagnosis and, above all, the treatment modality.

## Case description

This is a case of a 50-year-old female who was referred to the Unit of Minimally Invasive Endocrine Surgery of Kyanous Stavros Hospital for a total thyroidectomy due to a goiter. On presentation she complained of compressive symptoms caused by her enlarged thyroid. More specifically, she reported progressive dysphagia for the last two years and a new onset of positional inspiratory stridor. Her past medical history was otherwise insignificant; at that time, her only medication was thyrohormone to treat hypothyroidism. She is a non-smoker, and drinks alcohol socially. Presently, she cannot attribute any symptoms to the paracarotid mass identified during clinical examination.

**Figure 1:**
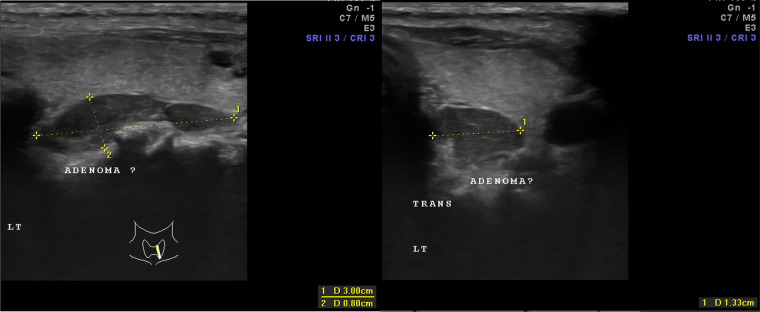
Sonography reports: in proximity to the posterior surface of the mid-portion of the left thyroid lobe, a well-circumscribed, flattened, hypoechoic, solid lesion was identified, measuring 30 × 8 × 13 mm. The mass exhibited significant peripheral and internal vascularity with a centripetal pattern, supplied by a vascular branch originating from the lower pole. Based on these imaging characteristics, the findings were primarily suggestive of a parathyroid adenoma.

**Figure 2:**
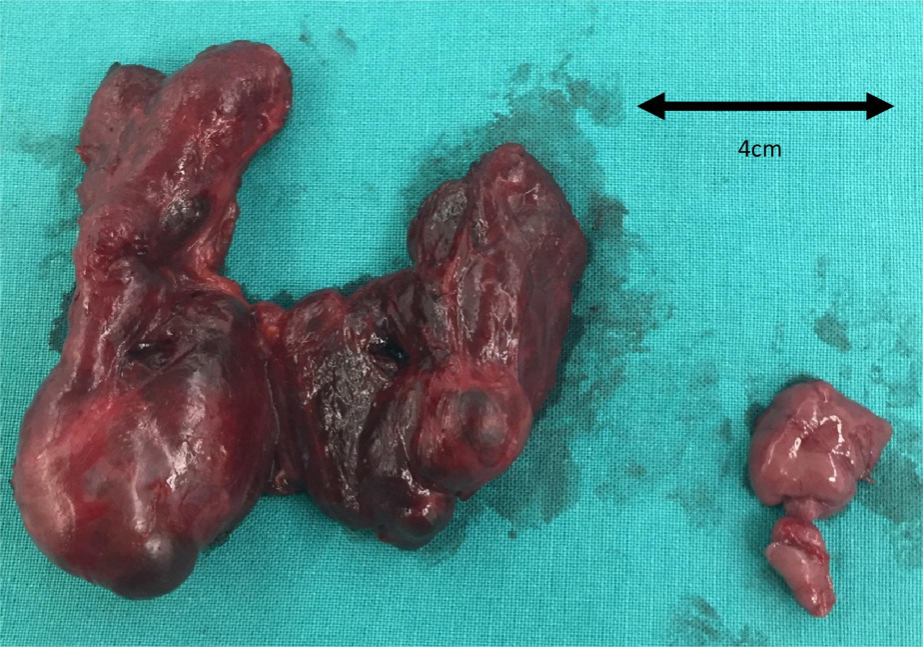
The surgical specimen of the total thyroidectomy (on the left side of the figure). There is a prominent thyroid nodule on the lower right lobe of the thyroid measuring 37 × 18 × 20 mm on final histology report. On the right side of the Figure is the paracarotid mass excised completely, without rupturing of the capsule. This mass was identified on histopathology as paracarotid rhabdomyoma.

Sonographic examination revealed a well-circumscribed soft tissue mass in the paracarotid space at Level III of the neck. This hypoechoic mass (measuring 30 × 8 × 13 mm on Ultrasound) appeared to have pericapsular and internal vascularity (Figure 1). The mass was in close proximity to the posterior margin of the left thyroid lobe and was characterised by the radiologist as a parathyroid adenoma (despite PTH levels being within the normal range). The mass did not present any signs of malignancy during sonography and, as reported, was asymptomatic and discovered incidentally. After discussion with the patient, a decision was made to proceed with total thyroidectomy and complete excision of the mass. Indeed, a total thyroidectomy was performed, including the surgical excision of this mass (Figure 2). The postoperative course was uneventful, and the patient was discharged the next day symptom-free.

**Figure 3:**
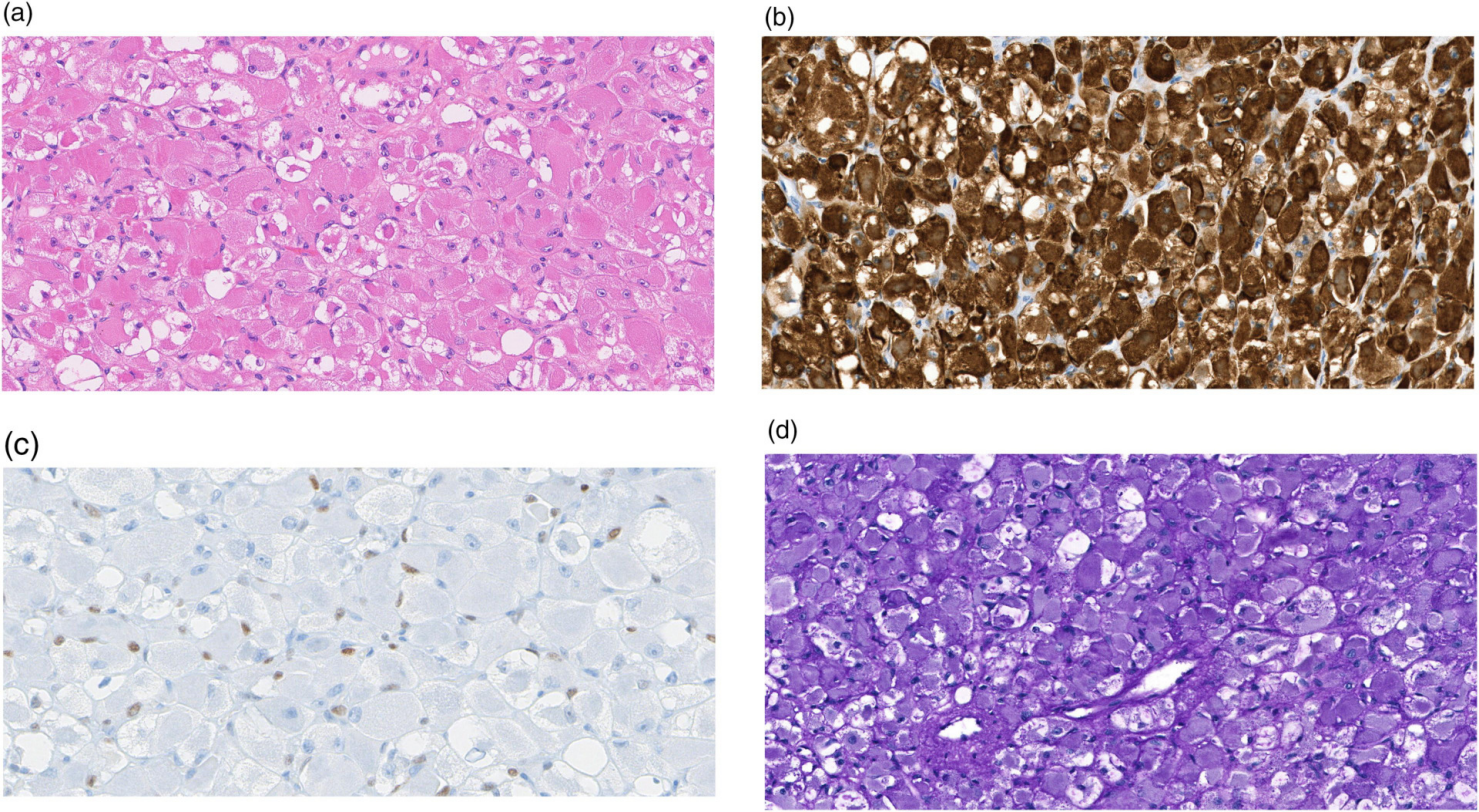
**(a)** Round or polygonal large cells with abundant eosinophilic fibrillar or granular cytoplasm and spider cells with vacuolated cytoplasm (HE x20). **(b)** Cells show diffuse cytoplasmic positivity for Desmin (Desmin x20). **(c)** Heterogenous nuclear positivity for MyoD1 (MyoD1 x40). **(d)** Tumor cells show PAS positivity (PAS x20).

Microscopic examination of the tissue sections revealed a neoplasm composed of medium-to-large polygonal cells characterized by distinct cell borders and abundant, granular eosinophilic cytoplasm. The nuclei were subround or oval-shaped, featuring prominent solitary nucleoli. No evidence of cellular atypia or mitotic activity was identified. The cells exhibited a solid architectural arrangement. Immunohistochemistry showed positivity for Desmin, MyoD1 and PAS, while S100 was negative (Figures 3a–d). These findings are characteristic of rhabdomyoma and confirmed the diagnosis, excluding other possibilities such as schwannoma, paraganglioma and parathyroid adenoma.

At her 5-year follow-up appointment, the patient was disease free with a well-controlled TSH value. Sonography five years postoperatively was negative for neck abnormalities.

## Discussion

Although the name rhabdomyoma was given by Zenker in 1864 (2), Von Recklinghausen first described the lesion in 1862 (4). Rhabdomyomas are extremely rare benign tumors originating from the mesenchyme of skeletal muscle. The presence of clonal structural chromosomal abnormalities in extracardiac adult rhabdomyomas confirms that they are true neoplasms, in contrast with the cardiac type, which are characterized as hamartomas (5). Cardiac rhabdomyomas are the most prevalent benign tumors in children and tend to regress spontaneously. Fortunately, no cases of malignant transformation have been described in the literature for either type. Fetal and adult subtypes are most frequently found in head and neck areas (oral cavity, parapharyngeal space, submandibular region, vocal cords, larynx, tongue, lip, nasopharynx, epiglottis) (6–10, 13, 14) with nearly 90% occuring in this region. Their topography is consistent with their embryologic origin, as they arise from the brachial musculature of the third and fourth brachial arches (11, 14). Differential diagnosis includes essentially any mass of the parapharyngeal space, namely neurogenic tumors (Schwannoma, Paraganglioma), salivary gland tumors (Pleomorphic adenoma), ectopic parathyroid gland/adenoma, ectopic thyroid tissue, and mesenchymal tumors such as lipomas or rhabdomyomas.

They usually appear as solitary, well-circumscribed, movable, asymptomatic round or polypoid nodules but very rarely can be multifocal. A short series of multifocal rhabdomyomas (27 cases) has been reported in the literature (12, 15, 16). Symptoms depend on the site and size of the lesions. Hoarseness, dysphagia, or respiratory distress due to laryngeal compression may be reported. Additionally, they can be present as a palpable neck mass, which may or may not be painful, as in the present case. A review of the relevant literature did not reveal any other case of rhabdomyoma in the paracarotid space.

Regarding diagnosis, imaging findings usually suggest a benign, non-invasive lesion but rhabdomyomas may mimic malignant tumors due to extension into surrounding areas (17). Thus, it is nearly impossible to establish a diagnosis preoperatively. In the current case, no further diagnostic modality was used as the suspicion of malignancy were low, and decision was made to perform an excision biopsy instead of an FNA. FNA is generally contraindicated for suspected paragangliomas for the risk of haemorrhage, and in cases of parathyroid adenoma, it may cause parathyromatosis. Should there not be a prior indication for surgery, further work-up would be indicated as per standard neck mass protocols. More specifically, a CTA or MRA of the neck could differentiate a carotid body tumor or a paraganglioma. The final diagnosis can only be confirmed by the cytologic and histologic findings. Interestingly, the initial ultrasonographic evaluation suggested a parathyroid adenoma. This was likely attributed to the lesion's proximity to the thyroid and its prominent centripetal vascularity. This underscores a potential diagnostic pitfall, as the hypervascular nature of rhabdomyomas can mimic the classic imaging features of endocrine or neurogenic tumors. Typical pathology reveals polygonal cells with eosinophilic granular cytoplasm and one or two peripherally located vesicular nuclei (9). The diagnosis of an adult-type rhabdomyoma can be confirmed by the strong positivity for desmin and myoglobin, coupled with the negativity for S100 and cytokeratins, indicating a skeletal muscle origin.

The recommended therapeutic strategy is complete surgical excision. The possibility of recurrence depends on the radicality of the excision. Recurrence or residual mass can occur in cases where the rhabdomyoma is multifocal. In those instsnces, reoperation is required. Fortunately, the tumor grows very slowly and no malignant transformation has been reported in the literature (1).

## Conclusion

Adult extracardiac rhabdomyomas are extremely rare skeletal muscle tumors. Although reported cases show they occur in various head and neck locations as solitary or multifocal lesions, this is, to the best of the authors’ knowledge, the first report of an adult rhabdomyoma in the paracarotid space. Definitive diagnosis was made by histology. Correct identification is crucial, as it may spare the patient aggressive treatment instead of a curative, limited excision.

## Data Availability

The raw data supporting the conclusions of this article will be made available by the authors, without undue reservation.
